# Salim and Quarraisha Abdool Karim: protecting young women, ending HIV

**DOI:** 10.2471/BLT.25.030325

**Published:** 2025-03-01

## Abstract

Salim ‘Slim’ and Quarraisha Abdool Karim talk to Gary Humphreys about their efforts to reduce the exposure of young women to infection with human immunodeficiency virus (HIV), the importance of perseverance, the opportunities presented by new long-lasting HIV therapies, and the need for unity in a challenging time.

*Q: You both grew up under apartheid in South Africa. How did that inform your development and later career choices?*

SAK: We’re both third-generation South Africans of Indian heritage. Growing up, I attended a school exclusively for Indian children, frequented beaches designated for “non-whites”, and rode buses reserved for black South Africans.

QAK: I grew up in Tongaat, a small village just outside Durban, with a primarily Indian population brought by the British as indentured labourers in 1860 to work in the sugarcane plantations. A commitment to education was one of the defining characteristics of my grandparents' generation, and with the support of merchants from India who had arrived with them, they raised money to bring teachers from Ceylon (now Sri Lanka) and establish schools in the village. I graduated from high school in 1976, the same year as the Soweto uprising in South Africa. That was the first time I developed a real understanding of apartheid beyond the daily restrictions I was used to, but it was at university that I was introduced to protest and resistance. As to my career choices, I always wanted to be a scientist, much to the consternation of my parents. They would have preferred me to be a doctor. They were not sure what scientists did.

SAK: I had a similar experience to Quarraisha, entering medical school in 1978 where I joined the anti-apartheid movement and was at the founding of the United Democratic Front, an anti-apartheid coalition. As a student activist, I focused my efforts on the violations of human rights and racial disparities in health. My first published article about 40 years ago was an analysis of apartheid's impact on health.

*Q: You are best known for what you have achieved in HIV research together. When did you first start to work together on the disease?*

SAK: We started really thinking about HIV in New York in 1987, when the disease was dominating public and academic discourse in the United States. I remember attending a pivotal lecture by Samuel Broder on AZT (azidothymidine, an antiretroviral drug that was the first medication approved for the treatment of HIV), demonstrating its efficacy in increasing CD4 (a type of white blood cell that plays a critical role in the immune system) counts in HIV patients. That lecture had a big impact on us both and contributed to our decision to commit to HIV research, which we did when we returned to South Africa in 1988.

QAK: New York was certainly pivotal, but I had already had some exposure to HIV/AIDS in South Africa in the early 1980s. A professor I was working with in Johannesburg had a son who had recently been diagnosed with HIV. Out of concern for other men in South Africa, he went on radio and television, specifically inviting men who have sex with men to come to the immunology department so that he could contribute to understanding the causes of infection and to provide support for those who might be sick. That was my first introduction to the disease and the young men who were getting it.

*Q: Which aspect of the pandemic did you focus on when you returned to South Africa in 1988?*

SAK: Initially we wanted to get a better understanding of the scale of the epidemic in Africa, and we launched a large community-based study in 1990 with over 5000 people. We felt that quantifying the scale of the problem and understanding its epidemiology was a good place to begin.

QAK: We got something of a head start because of the research I had been doing on South Africa’s Active Malaria Control Programme as part of my Master’s dissertation. The programme used community members to regularly visit a designated number of households to collect blood samples for malaria screening. I had the idea of using the system to conduct a population-based HIV prevalence survey. We ended up testing thousands of people from various demographics, and discovered an emerging HIV epidemic in the general population but also intriguing differences between young men and women.

*Q: Can you say more about that?*

SAK: It turned out that young men had almost no HIV, with rates staying low until about age 20, then increasing and peaking at age 30. For women, the pattern differed significantly; HIV was nearly non-existent until age 15 but surged and peaked by age 20. This led to the realization that the primary transmission route among these young women was not from their male peers but from older men in their 20s and 30s, often through transactional sex. As a result of the insights gained, we shifted our focus towards protecting these vulnerable young women, focusing on gels to protect the vagina.

*Q: Why do that rather than emphasizing the importance of condom use?*

QAK: In the early ’90s, the focus of the HIV response was on men who have sex with men and injecting drug use. The HIV prevention mantra focused on abstinence, being faithful, and using male condoms. I recall several women during interviews asking why scientists were not working on a woman-initiated method, pointing out that because of social norms, gender dynamics and economic dependence on male partners, women were rarely able to insist on condom use. We wanted to give them the power to protect themselves. But I have to be honest – when we first thought of developing a method that women could use, we had no idea what that journey would entail. In 1993, we initiated our HIV prevention research with a US company to develop a biodegradable film intended to serve as a vaginal barrier to block HIV transmission. The project failed, but it started an 18-year exploration of various vaginal products to prevent HIV, none of which succeeded! Finding viable products and navigating the many obstacles of conducting research with young women was a huge challenge. For example, the gels we were working with had to be effective against HIV but not harm the cells in the vagina. We had to work through these challenges while ensuring these methods were safe and prevented HIV. Initially, we worked with what was already available on the shelf. But at some point, we realized we needed to do better.

*Q: Eighteen years is a long time. Did you ever consider giving up?*

SAK: Never. People used to call us the "experts in failure" because we presented so many unsuccessful clinical trial results at international AIDS conferences, but along with five or six other groups worldwide, we persevered. In South Africa, this included setting up CAPRISA which we established in 2002 as part of a collaborative effort to bolster HIV research. It was there that we led the CAPRISA 004 trial, a landmark study conducted between 2007 and 2010, which evaluated the effectiveness of a vaginal gel containing 1% tenofovir, an antiretroviral drug, in preventing HIV transmission during heterosexual intercourse.

QAK: It was a huge breakthrough for us. The use of the tenofovir gel provided a 39% reduction in infection overall, and – among women who used the gel consistently – the reduction in HIV risk was even higher, up to 54%. It also demonstrated a 51% reduction in the risk of acquiring herpes simplex virus type 2, which often co-occurs with HIV. The findings were the first to demonstrate that a vaginal microbicide could effectively reduce HIV risk, providing a proof of concept for antiretroviral-based pre-exposure prophylaxis (PrEP).

SAK: I remember announcing our breakthrough at the 2010 Vienna AIDS Conference. It was met with a standing ovation, which was a welcome change after all the years of failure.

“Optimism, activism, advocacy, evidence and innovation can keep us moving forward.”

Quarraisha Abdool Karim

*Q: What has been the focus of your research since that breakthrough?*

QAK: We have continued to work on HIV and infectious diseases, taking account of the social dimensions of the epidemic while focusing on interventions empowering women.

SAK: Among our key discoveries is the role of genital inflammation in significantly elevating HIV infection risk. By studying cytokines and chemokines in the vagina, we identified an inflammatory profile that predisposes young women to infection. This inflammation brings CD4 cells closer to the vaginal wall's surface, making them more susceptible to HIV, which uses a co-receptor on activated CD4 cells to enter and infect the cells. Our most recent studies focus on an imbalance in the vaginal microbiome that contributes to an inflammatory profile that heightens HIV infection risk.

QAK: Prompted by insights derived from that research, we are now exploring potential therapeutic interventions. For example, in collaboration with the Vaginal Microbiome Research Consortium, we have isolated various strains of *Lactobacillus crispatus*. These have been formulated into capsules. We recently initiated a Phase I trial to test these vaginal capsules, aiming to beneficially modify the vaginal microbiome. We are also examining long-lasting pre-exposure prophylaxis (PrEP) and its implications for public health.

*Q: Long-lasting HIV therapeutics are very much in the headlines. What is the significance of their emergence for the pandemic?*

SAK: The emergence of long-acting antiretroviral formulations clearly represents a significant advance. Gilead’s lenacapavir is notable in this regard, being the first to have received regulatory approval. However, it is important to note that, to date, it has only received approval for the salvage therapy in ‘treatment-experienced’ individuals (people living with HIV who have previously received antiretroviral therapy but may have experienced treatment failure, resistance, or intolerance to one or more HIV medications). In clinical trials in women in Africa, lenacapavir was found to be 100% effective in preventing HIV. This is largely because, unlike traditional antiretrovirals, lenacapavir targets the viral capsid, disrupting multiple stages of the virus cycle. Resistance is likely to develop over time, especially if patients do not adhere to the six-month dosing schedule, which could lead to subtherapeutic drug levels and subsequent viral resistance. It is therefore important to temper expectations regarding the efficacy of these new drugs. Going forward, ensuring access to these drugs is going to be a major challenge, and will depend to a high degree on how they are priced and whether organizations like the Global Fund and The President’s Emergency Plan for AIDS Relief (PEPFAR) get behind them. And of course, assuming lenacapavir does turn out to be effective for PrEP and is priced affordably, we will still have to get it to the people who need it.

*Q: Which groups should be prioritized, in your view?*

QAK: The drug needs to reach those at highest risk, especially young girls. They are the demographic with the highest HIV incidence rates. And it must be done on a massive scale – potentially millions – to truly curb the public health threat of HIV by 2030. We believe strongly that the distribution should focus on hotspots (areas identified through routine analysis with high rates of new infections).

SAK: As challenging as all this may be, especially in the current funding environment, using lenacapavir as PrEP will not need to be indefinite. The risk of HIV acquisition decreases significantly once young women transition into more stable phases of life, such as marriage or long-term partnerships. Therefore, the preventive strategy might only need to cover a period of about four to eight years – roughly translating to 16 injections per individual. This finite treatment span could help manage the scale and cost of the programme.

QAK: Creating demand among young, healthy girls who may not perceive themselves at risk is crucial. And once the demand is established, the challenge will be managing the logistics of delivery without overwhelming the health services. Millions of healthy individuals seeking injections could significantly strain resources.

SAK: Implementation strategies could include integrating the administration of new long-lasting therapies with existing health campaigns, like vaccination drives, to streamline the process and reduce the burden on health facilities. In practical terms, we must develop innovative strategies that address both the creation of demand and the logistical challenges of delivering the treatment on such a large scale.

*Q: You have also been recognized for your contributions to capacity-building and bringing on the next generation of South Africa’s scientists. What have been your main contributions in that area?*

QAK: In 1994, after South Africa’s first democratic elections, I was asked to establish the country’s national AIDS programme. That transition – from science to policy – was a pivotal moment for us both, I think. We very quickly came to understand how ill-equipped we were to handle the unfolding epidemic, and the urgent need to build the research and programmatic capacity in South Africa. Together with South African-born epidemiologist and public health advocate Zena Stein and colleagues at the South African Medical Research Council, we secured a grant from the US National Institutes for Health’s Fogarty International Center to train South Africans in HIV research. Over its 22 years, this programme trained over 600 scientists in Lesotho, Namibia, South Africa and Swaziland. 

SAK: It is also worth mentioning that when we established CAPRISA in 2001, one of our key goals was training the next generation of scientists. We recruit talent from medical schools and support them through master's, doctoral and postdoctoral studies. We see this as the sustainability pathway – investing in people to build Africa’s scientific capacity. Our efforts extend beyond South Africa, and we facilitate exchange programmes and collaborations, both North-South and South-South.

“We must develop innovative strategies that address both the creation of demand and […] delivering the treatment.”

Salim Abdool Karim

*Q: Quarraisha, in 2022 you were elected President of The World Academy of Sciences (TWAS) which advances scientific research and capacity-building in the Global South. How significant was that for the work you do?*

QAK: TWAS is a great organization, with world-leading scientists as Fellows across the globe. The past two years as its President have been incredible – the opportunities, the interactions, and the opportunity to engage in complex global challenges with like-minded scientists trying to find solutions has been inspiring and motivating. COVID-19 reminded us of our shared vulnerabilities and interconnectedness. It reinforced the idea that we live on one planet and must work together. The beauty of our diversity – whether in thought, physical appearance, or geographic distribution – should be a strength we harness to create a sustainable future.

*Q: You were the seventh president to be elected, but only the first woman. To what extent does that reflect ongoing gender inequality in the public health space?*

*QAK: *TWAS has many highly talented female scientists, as reflected in membership since inception and currently in its gender-balanced Governing Council. While gender inequality remains a challenge in public health as in other science disciplines, I’ve been very fortunate. I’ve always had strong support – from my colleagues, my family, from Slim, and from my mentors, both men and women. But it certainly helps to stay focused on where you need to go rather than getting caught up in the distractions of the “pull-her-down” syndrome – what I jokingly refer to as the “other PhD.” Younger scientists today have a lot more role models of women in science including in leadership positions to look up to, and there are a lot more policies and programmes that more intentionally ensure gender equity that are contributing to this leadership gap being closed, but more remains to be done.

*Q: Given the current geopolitical and public funding situation, how optimistic are you with regard to achieving the SDG Target 3.3***** *of ending the AIDS epidemic by 2030?*

QAK: Right now, the world is faced with growing nationalism, racism and extremism. But I believe optimism, activism, advocacy, evidence and innovation can keep us moving forward. To reach our HIV goals, we need to make better use of the tools now available to us including through customized targeted approaches. Capsid-targeting therapeutics are a good example of this. They look exciting, but we have to be careful how we use them. You can’t just apply the same solution everywhere simply because you have one. Now is the time to think strategically about how to use these tools in a more targeted and effective way.

SAK: It is hard to overstate the importance of innovative approaches to implementing long-acting PrEP. We have to design strategies that not only get drugs, in sufficient volume, to the populations that need them, but to create demand for the drugs as they become available. That is going to be challenging.

